# Editorial: Microbiological aspects of non-conventional foods, ingredients and beverages

**DOI:** 10.3389/fmicb.2023.1340215

**Published:** 2023-12-12

**Authors:** Virgínia Farias Alves, Elaine C. P. De Martinis, Bruno Meireles Xavier, Uelinton Manoel Pinto

**Affiliations:** ^1^Faculdade de Farmácia, Universidade Federal de Goiás (UFG), Goiânia, Brazil; ^2^Ribeirão Preto School of Pharmaceutical Sciences, University of São Paulo (USP), São Paulo, Brazil; ^3^Department of Food Science, Cornell University, Geneva, NY, United States; ^4^Department of Food and Experimental Nutrition, Faculty of Pharmaceutical Sciences, Food Research Center, University of São Paulo (USP), São Paulo, Brazil

**Keywords:** non-conventional foods, microbial quality, microbial safety, novel foods, food safety

As consumers seek healthier diets that emphasize minimally processed, organic, and “natural” foods, it is crucial to ensure these healthier foods are safe and implement sustainable production methods while adhering to the principles of One Health. This approach seeks to maintain the delicate balance between humans, animals and the environment. Non-conventional foods (NCF) offer potential solutions to diversify dietary choices, enhance nutritional value, reduce the environmental footprint of human activities, and contribute to local and regional economies by minimizing food waste and promoting better health. NCF include unconventional food plants, foods derived from microorganisms, algae, insects, novel fermented substrates, among others. Despite recent advancements in the development of NCF, there is limited knowledge about their microbial safety hazards and spoilage characteristics. This Research Topic sheds light on the intricate microbiological aspects associated with NCF via seven articles, with fermentation being one of the most explored themes.

The work by Wang et al. on fermented cherry juice offers a promising pathway to overcome the challenges associated with these delicate fruits, which are prone to physical damage and fast spoilage. The authors emphasize a recent surge in production of sweet cherries in China, highlighting that fermentation could be a practical solution to increase shelf-life and expand the industrialization of these fruits. By fermenting cherry juice using the lactic acid bacteria (LAB) *Lactobacillus acidophilus, Lactobacillus plantarum*, currently *Lactiplantibacillus plantarum*, and *Lactobacillus rhamnosus* GG, currently *Lacticaseibacillus rhamnosus* (Zheng et al., 2020) the study explored the microbial growth as well as physicochemical and aroma characteristics of the product. All LAB strains demonstrated robust growth potential in the sweet cherry juice, with *L. acidophilus* and *L. plantarum* standing out for their acid production and viability. The study unveiled significant insights into the alterations in microbial metabolism, aroma profiles, and flavor transformation brought about by these LAB. The integration of electronic nose and headspace gas chromatography–ion mobility spectrometry contributed to a comprehensive understanding of biochemical changes. The study results may contribute to expanding the utilization of sweet cherries in the food industry and uncovered the flavor profiles of the product. Future research including sensory evaluation with consumers could further strengthen the current findings and substantiate their market potential.

Li et al. explored the genetic diversity of non-*Saccharomyces* yeasts associated with spontaneous fermentation of Cabernet Sauvignon wines from five subregions within Ningxia, a well-known China's viticulture region. There was a remarkable diversity of non-*Saccharomyces* yeasts and their potential role in shaping the organoleptic profile and quality of wine. By sequencing the 26S rRNA D1/D2 domain, the researchers identified 19 non-*Saccharomyces* yeast species belonging to 10 genera, with *Hanseniaspora uvarum, Candida zemplinina*, and *Metschnikowia pulcherrima* emerging as dominant species. The study delved into subspecies-level discrimination by Tandem repeat-tRNA fingerprinting (TRtRNA) analysis, which combines PCR amplification using two different primer sets, followed by gel electrophoresis. Depending on the primer pair, the 524 non-*Saccharomyces* isolates obtained in the study could be discriminated into 34 or 40 distinct TRtRNA profiles, suggesting a variable degree of discriminatory power when using the method. Using this approach, the study showed the existence of a diverse array of non-*Saccharomyces* yeast species in Ningxia, contributing to the pool of genetic resources essential for future strain development and application as starters in wine production. While still studying wines in China, Li and Hong investigated the effect of inoculation of autochthonous *Candida railenses* and *S. cerevisiae* on alcoholic fermentation and chemical and aromatic characteristics of Vidal blanc icewine. The obtained results indicated that *C. railenensis*, although with a lower capacity for alcoholic fermentation than *S. cerevisiae*, presents typical oenological properties since the strain positively influenced some of the chemical and aromatic parameters of Vidal blanc icewine, compared to icewine fermented only by *S. cerevisiae*. However, more research is needed to clarify the interaction between the tested yeast species during fermentation, and to verify the safety and metabolic pathways utilized by *C. railenensis*.

Fungi may contaminate foods causing spoilage and potentially affect their safety by producing mycotoxins. This problem is a concern for semi-moist pet foods which contain moisture levels of 25–35%. Deliephan et al. evaluated the effect of liquid smoke and other natural preservative alternative preparations on the shelf life of a model semi moist pet-food (moisture of 25–35%). The authors focused on the growth of naturally contaminating molds and of a toxigenic *Aspergillus flavus* strain inoculated into the food. The results showed liquid smoke preparations extended the product's shelf life and inhibited growth of *A. flavus*. Formulations containing a high carbonyl content exhibited the most promising results in the shelf life of the food. The authors point to the need for additional studies, particularly focusing on the impact of phenolic compounds and organic acids also present in the smoke preparations. While there is a growing preference for natural preservatives, the study did not evaluate consumer perception of liquid smoke added to pet foods. Still, more studies are needed to evaluate the effect of liquid smoke on palatability tests as these preservatives may compromise flavor. Following this line, to ensure the microbiological safety of palatable semi-moist pet-foods without the use of synthetic preservatives, Kiprotich et al. formulated a semi-moist dog-food, mimicking a commercial product, which underwent a challenge test with *A. flavus*. To inhibit fungal growth, fermented whey permeate (WPF), alone or in combination with citrus extract oil (CEX), was included in the formulation. Addition of WPF in quantities >1.0% (w/w), compromised the sensorial qualities of the product. However, the addition of CEX enhanced the antifungal efficacy and reduced the dose of WPF required to control *A. flavus*, indicating the technological feasibility of adding these natural constituents to pet food. Further studies are needed to identify the constituents and their concentration in WPF to clarify the mechanisms of fungal inhibition.

Although honey is an unconventional environment for microbial growth and survival, it contains a diverse microbial community, a large portion of which has not yet been recovered and identified by traditional culture methods. Xiong et al. utilized culture-independent methods to analyze the composition of microorganisms in honey from different sources, aiming to better understand the microbial dynamics of the hives, and its influence on the health and productivity of bees. Additionally, different physicochemical parameters of raw honey and their association with the microbial diversity were evaluated. Results show a greater variation in fungal communities than in bacterial communities and indicates a possible association between physicochemical properties of different types of honey and their microbiome. These results provide valuable information to expand our knowledge about microbial behavioral dynamics in honey. Additionally, this approach is particularly important in the context of One Health since the honey microbiota can be used as a bioindicator of ecological information about the microenvironment in which the microorganisms are hosted.

Liu et al. isolated a new strain of *Lactobacillus acidophilus* “C4” with potential to survive into the gut environment. The researchers tested the effect of the strain administration on mice with DDS-induced colitis, investigating possible mechanisms of action for the probiotic candidate. The results of the study indicated that *L. acidophilus* C4 was efficient in alleviating DSS-induced colitis, as in the presence of the tested strain there was a reduction in tissue pro-inflammatory cytokines and oxidative stress, increase in mucin production, expression of tight-junction proteins, as well as a greater production of short-chain fatty acids, effects that are associated with better regulation of intestinal barrier function. Additionally, a greater diversity and abundance of microorganisms was observed in the microbiota of mice when in the presence of *L. acidophilus* C4. The main contribution of Liu et al. was to elucidate the multiple mechanisms of *L. acidophilus* C4 action that contributed to ameliorate DSS-induced colitis, which involved mainly the repair of mucosal barriers and the recovery of intestinal microecological balance.

Altogether, this Research Topic of articles shows that the investigation of NCF remains a promising field of studies, with much to be discovered, especially regarding the microbial ecology in these products.

## Author contributions

VA: Conceptualization, Writing—review & editing. ED: Writing—review & editing. BX: Writing—original draft. UP: Conceptualization, Writing—review & editing.
